# Randomised Badger Culling Trial—no effects of widespread badger culling on tuberculosis in cattle: comment on Mills, Woodroffe and Donnelly (2024a, 2024b)

**DOI:** 10.1098/rsos.241609

**Published:** 2025-06-11

**Authors:** Paul R. Torgerson, Sonja Hartnack, Philip Rasmussen, Fraser I. Lewis, Peter O’Donnell, Thomas E. S. Langton

**Affiliations:** ^1^ Veterinary Epidemiology, University of Zurich, Zurich, Switzerland; ^2^ Department of Veterinary and Animal Sciences, University of Copenhagen, Copenhagen, Denmark; ^3^ Private Statistical Consultant, London, UK; ^4^ Department of Mathematics, University of Cambridge, Cambridge, UK; ^5^ Herpetofauna Consultants International, Halesworth, Suffolk, UK

**Keywords:** randomized, badger culling trial, statistical analysis, tuberculosis, cattle, epidemiology

## Abstract

Substantial problems remain with the Mills *et al*. (Mills CL, Woodroffe R, Donnelly CA. 2024 *R. Soc. Open Sci.* **11**, 240385. (doi:10.1098/rsos.240385) and Mills CL, Woodroffe R, Donnelly CA. 2024 *R. Soc. Open Sci.* **11**, 240386. (doi:10.1098/rsos.240386)) re-evaluations of the Randomised Badger Culling Trial (RBCT) data. These include: (i) analysis of counts rather than rates; (ii) overfitting; (iii) modelling of ‘confirmed’ herd breakdowns rather than total herd breakdowns; and (iv) errors in the Bayesian analysis of the data. More plausible approaches to RBCT data analysis, including analysis of total breakdown incidence, strongly suggest that there is no effect of proactive badger culling on the herd incidence of bovine tuberculosis, either within cull areas or in neighbouring areas.

## 1. Introduction

The Randomised Badger Culling Trial (RBCT) [1] proactive cull analysis, published in 2006 [2] suggested that culling of European badger *Meles meles* could affect the ‘confirmed’ (officially TB-free withdrawn; OTFW) incidence of tuberculosis (bTB) in cattle herds. Re-evaluations of RBCT data in 2024 have produced one view of culling having no effect on bTB herd incidence (rates) [3] and two where an effect is said to be supported [4,5].

## 2. Effect of proactive badger culling on incidence of bovine tuberculosis in cattle

Poisson regression analysis of RBCT data concluded that culling badgers can reduce bTB herd incidence in cattle [2]. Table 1 presents the findings and appraisal analytics for the model used in 2006, and three other models, to demonstrate key statistical issues (further information is given in electronic supplementary materials, S1–S3).

**Table 1.** A selection of the frequentist models analysed in [3,4]. This compares the original model from [2] (and its equivalent in generalized Poisson form). Mills et al. [4] claim to be a robust interpretation of the RBCT results and ‘strongly’ support an effect of badger culling upon bTB herd incidence. The present study compares this with those models with the lowest Akaike information criterion (AICc) values which provide no support for an effect of culling.

models from Mills *et al*.	equivalent in Torgerson *et al*.	structure	estimated effect of culling (95% CI)	BIC	AICc	LOOCV RMSE	number of estimated parameters	parameter for exposure (95% CI)	comments
1	1	original Poisson generalized linear model	−18.7% (−29.5%, −6.2%)	155.2	203.0	9.97	13	0.05 (−0.44, 0.53	very high AICc indicates poor predictive powers. Exposure parameter not significantly different from 0 indicating implausibility.
3	3	original generalized linear model in generalized Poisson form	−18.7% (−24.6%, −12.3%)	147.1	217.2	9.95	13+1^a^	0.04 (−0.21, 0.30)	very high AICc indicates poor predictive powers. Exposure parameter not significantly different from 0 indicating implausibility.
4	3a	generalized Poisson (without any culling effect)	none	161.7	160.4	10.93	3+1^a^	0.66 (0.33, 0.98)	low AICc, plausible parameter for exposure parameter
8	4d	generalized Poisson with herd-years-at-risk covariate without any culling effect	none	155.5	154.2	8.81	3+1^a^	0.51 (0.32, 0.70)	lowest AICc of all the Poisson family models, indicating the best predictive power. Lowest LOOCV RSME indicating generalizability. Exposure parameter is significantly higher than 0, indicating plausibility, although as it is somewhat less than 1, it might indicate the offset model would be more appropriate.
null		generalized Poisson model with no predictors	NA	178.1	176.8	17.29	1+1^a^	NA	null intercept only model (when correcting for overdispersion) has lower AICc than models 1 and 3

^a^ Additional parameter in generalized Poisson models to model overdispersion.

### 2.1. Incidence rates and counts

Counts are unadjusted number of herd breakdowns without any reference to the number of herds or time of observation in the sample. Rates are number of herd breakdowns per unit herd, per unit time. RBCT data was compared between culled and control areas using counts, and Mills *et al.* [4] claimed this approach (Model 1) was ‘robust’, passing many (but not all) of the statistical appraisal citeria. Model 1 used number of herds as an explanatory variable. Mills *et al.* [4] indicate: ‘Another possible option for a Poisson regression model is the usage of an offset variable which enables modelling the count variable … as a rate, and the usage of an offset variable means that the corresponding regression coefficient is constrained to be 1’. However, in Model 1, with the unconstrained variable of log(number of herds), the parameter value is 0.04 (close to 0), indicating herd breakdowns do not vary with number of herds (i.e. count remains the same regardless of sample size). This approach is biologically implausible. Mills *et al.* [4] states: ‘Alternatively, assuming an offset variable not be supported by evidence (i.e. the number of events may increase non-proportionally with the population at risk) one could use an unconstrained regression coefficient and hence, instead of assuming the slope for the variable is exactly 1, the slope parameter is estimated’. In the purported ‘robust’ Model 1 there is no ‘non proportional increase in the counts with increase sample size’. There is no increase at all, demonstrating model misspecification. When managed by using an offset variable (Model 2) and corrected for overdispersion, treatment effect of culling becomes non-significant. Alternatively, removing triplet, the coefficient of the exposure variable becomes 0.64, with the upper confidence interval close to 1. Biological plausibility is shown by increase in counts with sample size. However, there is no significant effect of culling. Similar issues apply to post-trial period analyses (electronic supplementary material, S2 and S3). The mathematical derivation of the offset is explained in our previous manuscript on this issue [3].

### 2.2. Model appraisal and diagnostics

Mills *et al.* [4,5] use small sample size Akaike information criterion (AICc), Bayesian information criterion (BIC), leave-one-out cross validation (LOOCV) and posterior predictive checks (PPC) for model diagnostics. The optimal model published in Torgerson *et al.* [3] has substantially lower AICc than Model 1 and better LOOCV values (table 1). However, Mills *et al.* dismissed AICc as useful only as a ‘predictive diagnostic’ but would rather use the BIC as it gives ‘better performance for goodness of fit’; thus defending Model 1 as ‘robust’*.* Although a predictive model would be a better outcome for an experiment informing culling policy, the differences in the BIC between the two models are inconsequential. Mills *et al.* [5] state that LOOCV approximates to ‘model generalizability’. Therefore, LOOCV is preferable, as bias is negligible [6].

Mills *et al.* [4] claim that a visual PPC indicates that posterior predictive distribution of Model 1 resembles the observed data, and in Model 8 the PPC implies potential misfit due to ‘systematic discrepancies between model-predicted data and confirmed incidence’. A visual PPC can appear markedly different between simulations, often with no obvious difference between two competing models (electronic supplementary material, S2 and S3). PPC also uses data twice [7] by first estimating parameters from the observations, then by checking how close the observations are to model predictions.

Similar issues in model diagnostics can be identified in the models, from the ‘time to follow up’ models (electronic supplementary material).

### 2.3. Overfitting

Overfitting explains why Model 1, with 13 free parameters, has generally poorer diagnostics (i.e. AICc, LOOCV) compared with Model 8 which has just four free parameters. Although Model 1 satisfies the *p* < *n* rule (*p* is the number of parameters and *n* is the number of data points) and avoids saturation (although not by much), overfitting issues remain. Model 1 also has a higher AICc than the null, intercept only (generalized Poisson) model. It has been suggested that for each parameter, there should be at least five data points and a solution to the overfitting problem is to combine dichotomous variables into a continuous variable [8]. Model 8 combines all 10 dichotomous variables of Triplet into a single continuous one, of years at risk (electronic supplementary material). The elimination of ‘Triplet’ may remove any hidden effects possibly due to collinearity with number of herds, as well as substantially reducing the number of covariates. Hence, it largely solves the issue of overfitting.

### 2.4. Modern interpretation of comparative intradermal skin test (**single intradermal comparative cervical tuberculin**) reactors

Diagnostic tests rarely have *both* the sensitivity and specificity of 100%. Epidemiological theory demands that analyses should include the diagnostic error of the test [9], for example, modelling the COVID-19 pandemic [10]. The specificity of single intradermal comparative cervical tuberculin (SICCT), used in the RBCT is 100% [11], but with low sensitivity. Mills *et al.* [4] avoid the key issue of whether ‘unconfirmed’ breakdowns (officially TB-free status; OTFS) should have been included. These herds were infected with bTB according to test specificity. The inability to confirm disease status was due to poor sensitivity of post-mortem: 46% by meat inspection and 76% by detailed necropsy [11].

‘Unconfirmed’ reactor cattle, are essential in the analysis of the RBCT experiment, designed to monitor the effects of interventions. There was no evidence of a culling effect on total herd breakdowns (‘confirmed’ and ‘unconfirmed’ together) either in 2006 [2] or more recently [3]. All modelling approaches indicate that badger culling has no effect on total herd breakdown incidence is the robust RBCT conclusion.

Based on the RBCT, Donnelly and Woodroffe, predicted that ‘better prospects for the control of cattle TB are offered by badger populations that are either reduced by more than 70% or left undisturbed—and potentially vaccinated’ [12]. However, Mills *et al.* [4] set aside their own analysis as inferior in ‘predictive accuracy’. The models with best predictive accuracy suggest no overall reduction in bTB from 70% culling.

## 3. Bayesian analysis

Mills *et al.* [4,5] purportedly support their conclusions with Bayesian models equivalent to the Bayesian models in Torgerson *et al.* [3]. However, important issues invalidate these models presented by Mills *et al.* They claim, in error, that several models are a direct comparison to those of Torgerson *et al.* When examining the code and the effect size of culling it becomes apparent that the models are not the same. Other models claim to use an offset but due to coding errors the offset is omitted. The issues of the Mills *et al.* Bayesian models are summarized in table 2.

**Table 2.** A selection of Bayesian models for confirmed bTB herd breakdowns from initial cull until 4 September 2005 within proactive culling/control areas of the RBCT experiment. The original, frequentist Poisson generalized linear model (GLM) used in Donnelly *et al*. [2] was respecified in the Bayesian paradigm in the initial preprint, subsequently published paper by Torgerson *et al.* [3]. However, the Model rs was not specified by a negative binomial likelihood, although [4] reported it as Model a1 in error. Other errors and inconsistencies between those reported in [4] and Torgerson *et al.* [3] initial preprint and paper (electronic supplementary material, S2 and S3) for the Bayesian analysis paradigm (table 2a,b in [4]) are summarized here. In total of the eight models specified in table 2a,b of [4], five had errors. Hence, the analysis cannot be relied upon for *any* of the Bayesian analyses in [4].

model from [4]	model from [3]	issue of concern	notes	conclusions
a.1.	rs^a^	a.1 is not a correct representation of model rs. a.1. Uses negative binomial family model with a strongly informative exponential prior distribution for the reciprocal dispersion parameter	rs model was the Bayesian version of the model used in nature. As a Poisson, it has no dispersion parameter	all comparisons between a.1. and rs are invalid
a.2.		uses a negative binomial family with less tightly constrained prior for auxiliary parameter	now gives similar results to rs as converges to Poisson due to high reciprocal dispersion parameter	
b.1.	rs1B^a^		effect size and LOO values align with code given	
b.2.			effect size and LOO values align with code given	
c.1.	rs1aB^a^	no offset^b^ used due to coding error	effect size and goodness of fit are not reported correctly in table 2a	all comparisons between models c.1. and rs1aB are invalid. All subsequent analysis and conclusions based on model c.1. are invalid
c.2.		no offset^b^ used due to coding error	effect size and goodness of fit are not reported correctly in table 2a	all subsequent analysis and conclusions based on model c.2. are invalid
d.1.	rs2B		aligns with model rs2B	
e		statistical code given is not consistent with results reported	reported effect size does not align with results from code given.	all subsequent analysis and conclusions based on model e are invalid, including fig. 1 in the text of Mills *et al*. [3]

^a^ In the supplementary material of [4], the three models rs, rs1B and rs1aB and rs2B from [3] were reported to be the same models as a.1., b.1., c.1. and d.1., respectively.

^b^ Offset was reported in table 2b for models c.1 and c.2, but due to a coding error, the offset was not used. The effect sizes and LOO ELPG (expected log pointwise predictive density) values reported are consistent with no offset being used.

The Bayesian model selection techniques, such as Bayes factors, account for the complexity of the model (compare model rs2B with rs in Torgerson *et al.* [3], for example). Models without Triplet and Treatment as covariates are better supported by orders of magnitude, compared with those including these explanatory variables. Thus, there is no effect of culling on bTB herd incidence rates. Leaving aside the errors (table 2), Mills *et al.* made no comparison with suitable null models.

## 4. Statistical audit

A reviewer of the Mills *et al.* papers requested further details of the statistical audit of the RBCT [13], but this was not pursued. The RBCT statistical auditor had stated, *‘*to some extent, the number of triplets and the years of observation are interchangeable’ [14]. This interchange is implemented when herd years at risk is used as an explanatory or offset variable. This implementation fails to demonstrate a culling effect (see models 8 and 4d in table 1 and electronic supplementary material, S2). If this alternative analysis was done in the RBCT, it was not reported. The auditor also recommended that the primary analysis should consist of ‘log number of breakdowns per trial area in the form: Treatments; Triplets; Treatment x Triplets; Poisson error’. An interactive term of Treatment x Triplet leads to a saturated model (at least 20 predictors for 20 data points); evidence that the ‘independent audit*’* was inadequate. An interaction analysed by replacing Triplet with herd years at risk avoids saturation but demonstrates no evidence for a culling effect (electronic supplementary material, S2). It is important that trials include an audit that is open and transparent.

## 5. Neighbouring area study

### 5.1. Frequentist approach

The statistical concerns are similar to those issues found in [4], as summarized in table 3 and electronic supplementary material, S2 and S3. Mills *et al.* [5] also found no cull effect on total herd breakdowns, in accordance with Torgerson *et al.* [3].

**Table 3.** A selection of the frequentist models analysed in [3,5] on ‘neighbouring areas’. This compares the original model from the 2006 study [2] (and its equivalent in generalized Poisson form). This is the model that [5] claim to be a robust interpretation of the RBCT results and that ‘strongly’ supports a culling effect. We compare this with those models with the lowest AICc values, which provide no support for an effect of culling.

models from Mills *et al*.	equivalent in Torgerson *et al.*	structure	estimated effect of culling (95% CI)	BIC	AICc	LOOCV RMSE	number of estimated parameters^a^	parameter for exposure (95% CI)	comments
1	6	original Poisson generalized linear model	28.8% (5.7%, 57.2%)	148.3	196.0	8.07	13^b^	0.10 (−0.34,0.55)	very high AICc indicates poor predictive powers. Exposure parameter not significantly different from 0 indicating implausibility.
3		original generalized linear model in generalized Poisson form	28.5% (14.2%, 44.7%)	143.7	213.8^c^	8.05	13+1^b^	0.11 (−0.16, 0.38)	very high AICc indicates poor predictive powers. Exposure parameter not significantly different from 0 indicating implausibility.
4		generalized Poisson (without any culling effect)	none	154.5	153.2	8.26	3+1^b^	0.73 (0.33, 1.14)	low AICc, plausible parameter for exposure parameter
5	6a	generalized Poisson with herd-years-at-risk offset	10% (−9.1%, 33.0%)	163.0	210.7	12.07	12+1^b^	1	high AICc and BIC, high LOOCV RSME, culling effect not sigificant
6		generalized Poisson with herd-years-at-risk offset and without a culling effect	none	151.9	150.4	8.00	2+1^b^	1	low AICc and BIC, low LOOCV RSME indicating generalizability. Offset fixes exposure parameter to 1.
8		generalized Poisson with herd-years-at-risk covariate without a culling effect	none	145.8	144.5	6.86	3+1^b^	0.61 (0.39, 0.84)	lowest AICc of all the Poisson family models, indicating the best predictive power. Lowest LOOCV RSME indicating generalizability. BIC indicates better ‘goodness of fit’ than model 1. Exposure parameter is credible, although less than 1.
11	6b	generalized Poisson model with no predictors and with herd-years-at-risk offset	NA	151.2	149.9	8.15	1+1^b^	1	null model with exposure parameter fixed (when correcting for overdispersion) has lower AICc than models 1 and 3. No outliers (electronic supplementary material). Posterior predictive check does not always give systematic discrepancies between the model-based predictions and the confirmed incidence (electronic supplementary material).

^a^ Includes intercept.

^b^ Additional parameters are the intercept and in generalized Poisson models a parameter for overdispersion.

^c^ This was mistakenly reported as 217.2 in [5].

### 5.2. Bayesian approach

The issues in the Bayesian approach in [5] are similar to those in [4] and are summarized in table 4. It is worth noting that model d.2 (one model that was correctly coded) by their own analysis, ‘does not contain the implausibly large synthetic model-based predictions; furthermore, the estimated out-of-sample predictive accuracy (measured by LOO ELPD[expected log pointwise predictive density]) and, hence, the generalizability of the model are improved’. Nevertheless, Mills et al. dismiss it because it ‘does not account for any effect of culling’. However, modelled incidence is independent of culling (i.e. culling has no effect). Furthermore, this model is supported decisively by Bayes factors [15] compared with the RBCT model.

**Table 4.** A range of Bayesian models for confirmed bTB herd breakdowns from initial RBCT cull until 4 September 2005, on ‘neighbouring areas’ . The original, frequentist Poisson generalized linear model (GLM) used in [2] was respecified in the Bayesian paradigm in the initial preprint subsequently published by Torgerson *et al.* [3]. However, the Model rs in Torgerson *et al.* [3] was not specified by a negative binomial likelihood, although [5] reported it as Model a.1. in error. Other errors and inconsistencies between those reported in [5] and Torgerson *et al.* [3] for the Bayesian analysis paradigm (table 2a,b in [5] are summarized here). In total of the 8 models specified in table 2a,b of [5], 5 had errors. Hence, the analysis cannot be relied upon for any of the Bayesian analysis in [5].

model from [5]	estimated effect of culling (95% CI)	LOO EPLD	issue of concern	notes	conclusions
a.1 (varying intercepts for triplets and covariates of culling effect, historical 3 year incidence and baseline herds at risk). Claimed to be model rs^a^ from Torgerson *et al*.	31.3% (−29.4%, 160.9%)		a.1 is not a correct representation of model rs^a^. a.1. uses negative binomial family model with a strongly informative exponential prior distribution for the reciprocal dispersion parameter.	rs^a^ model was the Bayesian version of the model used in nature. As a Poisson, it has no dispersion parameter.	any comparisons between a.1. and rs^a^ are invalid
a.2 (a.1 improved)	24.5% (−3.6%, 69.9%)	−74.0	uses a negative binomial family with less tightly constrain prior for auxiliary parameter	now gives similar results to rs^a^ as it converges to Poisson due to high reciprocal dispersion parameter	
b.1 (no varying intercepts for triplets)	25.6% (−19.4%, 97.8%)	−74.9		outputs align with code	
b.2.(b.1 improved)	24.6% (−10.4%, 77.4%)			outputs align with code	
c.1 (using herd-years-at-risk as an offset and no varying intercepts for triplets)^a^	25.8% (−19.5%, 97.2%)		no offset^b^ used due to coding error	effect size and LOO values align with a non-offset model	all conclusions arising from c.1. model assuming the offset are invalid
c.2 (c.1 improved)			no offset^b^ used due to coding error	effect size aligns with a non-offset model	all conclusions arising from c.2. model assuming the offset are invalid
d.1(no culling effect)^a^	none		exposure variable 0.59 (0.20, 1.00)		
d.2 (d.1 improved)	none		exposure variable 0.58 (0.31, 0.83)	while the improved model structure does not enable direct, probabilistic inferences about the size of the effect of proactive culling, the lack of any discernible model diagnostic flaws is indicative of the appropriateness of the model (which does not account for any effect of culling)	model diagnostics suggest this model has the most support
e (Poisson with baseline herds at risk as an offset)	26.6% (4.3%, 52.8%)	−70.0	no offset^b^ used due to coding error	effect size and LOO values align with a non-offset model. This is effectively the nature model in Bayesian form	all conclusions arising from e model assuming the offset are invalid
e.1. (offset correctly coded)	9.7% (8.2%, 30.3%)	−85.5	model e with offset correctly coded	now substantial portion of probability density lies below 0.	

^a^ In the supplementary material of [5] the models a.1., c.1. and d.1. were reported to be the same models as rs, rs1aB and rs2B respectively from [3].

^b^ Offset was reported in table 2b for models c.1 and c.2 and e, but due to a coding error, the offset was not used. The effect sizes and LOO ELPG values reported are consistent with no offset being used.

## 6. Conclusions

In the frequentist approach to the examination of the 2006 RBCT data [2], there are three main issues: (i) analysis of counts rather than rates; (ii) overfitting; and (iii) modelling of ‘confirmed’ breakdowns rather than total (OTFW + OTFS) breakdowns. Mills *et al.* [4,5] fail to address these issues adequately and the Bayesian analysis has too many errors for a full critique.

Brewer *et al.* [16] have written about the dangers of relying on theoretical distinctions between AIC/AICc and BIC. Nevertheless, Mills *et al.* [4,5] state ‘… a wide array of statistical techniques and study periods allows us to make robust conclusions regarding the effects of proactive badger culling which are informed by consistent scientific evidence from trial data, irrespective of which approach to statistical inference is taken’. This is demonstrably untrue. The analysis of *‘*confirmed breakdowns’ (OTFW) show that results are highly dependent on the approach to statistical inference and information criteria used. However, absence of any cull effect on the incidence of bTB, when total breakdowns are considered, is robust, irrespective of statistical method.

The position of Donnelly that [17] ‘the suggestion of requiring independent replication of specific statistical analyses as a general check before publication seems not merely unnecessary but a misuse of relatively scarce expertise’, needs revisiting. The present case underlines the obligation not only for rigorous checks of statistical analysis but also for validation of the statistical models and assumptions used within submitted manuscripts to verify them. Accordingly, a very substantial number of publications that rest extensively or completely on RBCT statistical analyses may require major qualification or retraction. The justification for lethal control of badgers to date appears to have been based upon basic statistical oversight.

## Data Availability

All data and statistical code are included in the supplementary material [18]. Also we refer to three papers extensively, which also have code and data relevant to this manuscript. These manuscripts can be found at [3–5].
